# Aldosterone inhibits Dot1l expression in guinea pig cochlea

**DOI:** 10.1186/s40001-023-00994-y

**Published:** 2023-01-13

**Authors:** Shixun Zhong, Biyun Zhang, Li Qin, Qianying Wang, Xiaoli Luo

**Affiliations:** 1 Department of Otolaryngology, The First Affiliated Hospital of Chongqing Medical University, 1 Youyi Road, Yuzhong District, Chongqing, 400016 China; 2 Department of Otolaryngology, Mianyang Central Hospital, Mianyang, China; 3 Department of Otolaryngology, Chongqing Traditional Chinese Medicine Hospital, Chongqing, China

**Keywords:** Dot1l, Aldosterone, Endolymphatic hydrops, Meniere’s disease

## Abstract

**Background:**

Aldosterone relieves transcriptional repression of epithelial sodium channel (ENaC) by inhibiting Dot1a and Af9 expression and their interaction with ENaC promoter in various tissues. Expressions of ENaC and Af9 in inner ear have been identified. However, it is not known how Dot1l is regulated by aldosterone in inner ear.

**Methods:**

Twenty-eight adult guinea pigs were randomly divided into the control group and treatment group. Aldosterone 1 mg/kg/d was injected intraperitoneally in the treatment group and saline in the control group for 7 days. Animals were killed 1 month later following auditory brainstem response examination. Histomorphology of cochlea was detected with hematoxylin–eosin staining, and Dot1l expression was examined with immunohistochemistry and Western blot.

**Results:**

There was no significant difference in ABR thresholds before and after injection of aldosterone or saline in either group. Endolymphatic hydrops was found in 75% of animals in the treatment group. Dot1l was found in both groups in the stria vascularis, Reissner’s membrane, spiral limbus, organ of Corti and spiral ligament. Dot1l expression in the treatment group was decreased by aldosterone.

**Conclusions:**

Dot1l in guinea pig cochlea is inhibited by aldosterone with induction of endolymphatic hydrops. Dot1l may be closely related to endolymph regulation by aldosterone and to pathogenesis of Meniere’s disease.

## Background

Endolymphatic hydrops (EH), the pathological basis of Meniere’s disease, may result from abnormal Na^+^ and K^+^ metabolism. There are a lot of studies on K^+^ metabolic mechanism while a few on Na^+^ metabolism in endolymph up to date.

Epithelial Na^+^ channel (ENaC) is the rate-limiting step of Na^+^ reabsorption in renal tubular epithelial cell, colon and respiratory epithelium extensively. Our previous papers have shown ENaC expression in cochlea of guinea pig and rat [1, 2]. Studies on kidney indicate that ENaC expression is regulated by histone H3 lysine 79 (H3K79) methyltransferase disruptor of telomeric silencing 1 (Dot1). Dot1 is primarily identified in *Saccharomyces cerevisiae*, and Dot1l (Dot1-like) is its homogenic counterpart in mammal [3]. Further studies have identified that Dot1/Dot1l is a histone methyltransferase [4, 5]. Dot1a, Dot1l alternative splice variant a, binds to ENaC promoter via forming a Dot1a–Af9 complex, hypermethylates histone H3K79 at the endogenous ENaC promoter, and represses ENaC transcription [6]. Aldosterone relieves aforementioned repression by inhibiting the Dot1a and Af9 expression and their interaction with ENaC promoter [7]. Our previous study has demonstrated Af9 expression in guinea pig cochlea, and showed that aldosterone downregulates Af9 expression while induces EH [8]. Downregulated Af9 expression may inhibit Dot1a binding to ENaC promoter. However, it is still unknown whether Dot1l expression is regulated by aldosterone in inner ear.

The present study reports for the first time the regulation of Dot1l expression by aldosterone in guinea pig cochlea to investigate the possible role of Dot1l in endolymph regulation and thus in pathogenesis of MD.

## Methods

### Animals

Twenty-eight adult guinea pigs weighing 250–300 g, with normal Preyer’s reflex, were divided randomly into two groups, 14 in each group. Aldosterone (A9477, Sigma, American) 1 mg/kg/d was injected intraperitoneally in animals of the treatment group and 25 μl saline was injected in those of the control group for 7 days [8, 9]. Animals were killed under deep anesthesia by intraperitoneal pentobarbital sodium solution (40 mg/kg) 1 month later. Six animals in each group were used for histopathology and immunohistochemistry, and the others for Western blot analysis. The animals were provided by the experimental animal research center of Chongqing Medical University. All procedures concerning the animals use and care were approved by Chongqing Medical University Animal Use Committee.

### ABR measurements

Hearing was measured by auditory brainstem response (ABR) as described by our previous article [8]. Briefly, tests were conducted with an Intelligent Hearing Systems device (Smart EP, Intelligent Hearing Company, USA) in a soundproof and electrically shielded room. Animals were anesthetized deeply followed by inserting electrodes subcutaneously. The reference electrode was inserted on the measured ear, the ground electrode on the contralateral ear, and the record electrode under the scalp. A total of 1024 responses to click stimulus signal band-pass filtered at 100–3000 Hz were amplified and averaged. The level of testing signal was initially decreased in 10-dB steps from 90 dB SPL and then in 5-dB steps to search for the threshold, which was defined as the lowest level at which a detectable wave III response was elicited and repeated.

### Pathomorphology and immunohistochemistry of cochleas

The animals were anesthetized and then transcardially perfused with saline followed by fixation with 4% paraformaldehyde in 0.1 M phosphate-buffered saline (PBS) till the necks were stiff. The cochleas were fixed in 4% paraformaldehyde for 24 h and decalcified in 10% ethylene diamine tetraacetic acid (EDTA) for 1 month. The cochleas were dehydrated in alcohol with gradient concentrations and embedded in paraffin routinely and sectioned into 4-μm-thick sections across the modiolus.

For the pathomorphological examination, the tissue sections were stained with hematoxylin and eosin (HE), rinsed, dehydrated, coverslipped and detected with light microscope (Olympus, Japan). The endolymphatic hydrops was quantitatively analyzed by calculating the increase ratios (IR) using the formula IR = area A/area B × 100% [10, 11]. A represents the bulging part of scala media, while B represents idealized scala media area with straight Reissner’s membrane. Endolymphatic hydrops was achieved once IR was greater than 10%.

Immunohistochemical procedures were performed according to the protocol of the rabbit IgG SABC immunohistochemical staining kit (SA1022, Boster, China). Sections were deparaffinized and rehydrated routinely and then restored with 0.05% pancreatic enzymes (AR1007, Boster, China) for 20 min at 37 ℃ and blocked by 5% BSA for 10 min at room temperature. Subsequently, the sections were orderly incubated with rabbit anti-Dot1l antibody (1:100, ab64077, Abcam, UK) overnight at 4 ℃, biotin labeled goat anti-rabbit IgG and SABC for 30 min at 37 ℃, respectively. The Dot1l localization was revealed with DAB color reagent peroxidase substrate (AR1022, Boster, China). The nuclei were counter-stained with hematoxylin. The primary antibody for negative control was replaced by PBS.

### Western blot

Proteins of cochleas were extracted with cell lysis buffer for Western (P0013, Beyotime, China). Protein concentrations were measured with the BCA Protein Assay Kit (P0013, Beyotime, China). Total protein was separated by 8% SDS-PAGE electrophoresis and transferred to PVDF membranes. The membranes were blocked with 5% BSA and incubated with rabbit anti-Dot1l antibody (1:200, ab64077, Abcam, UK) or anti-GAPDH antibody (1:1000, AF1186, Beyotime, China) overnight at 4 ℃ and subsequent HRP-labeled Goat Anti-Rabbit IgG (1:1000, A0208, Beyotime, China) for 1 h at 37 ℃. Immunolabeled bands reacting with a chemiluminescent substrate BeyoECL Star (P0018A, Beyotime, China) were detected and quantified by FUSION-FX7 Spectra (Vilber, France).

### Statistical analysis

All data (mean ± SD) were analyzed with SPSS 20.0 (Chicago, USA) and GraphPad Prism 6.0 (California, USA). ABR thresholds were analyzed with paired t-test, Western blot and IR with independent samples *t*-test. *P* < 0.05 was considered statistically significant.

## Results

### Auditory function

In order to identify whether the aldosterone injected impairs hearing, hearing threshold was measured with ABR. Our results showed that there was no significant difference in ABR thresholds before and after injection of aldosterone or saline in either group (Table 1). It indicated that the hearing was not affected by aldosterone administration.

**Table 1:** The ABR thresholds of all animals (*n* = 28)

Group	*n*	ABR threshold before treatment (dB SPL)	ABR threshold after treatment (dB SPL)	*P*
The control group	14	16.79 ± 4.64	18.57 ± 6.02	*P* = 0.27
The treatment group	14	20.03 ± 4.80	20.71 ± 4.74	*P* = 0.77

The ABR thresholds before and after injection of aldosterone or saline between two groups were not significantly different

### Endolymphatic hydrops induced by aldosterone

**Fig. 1:**
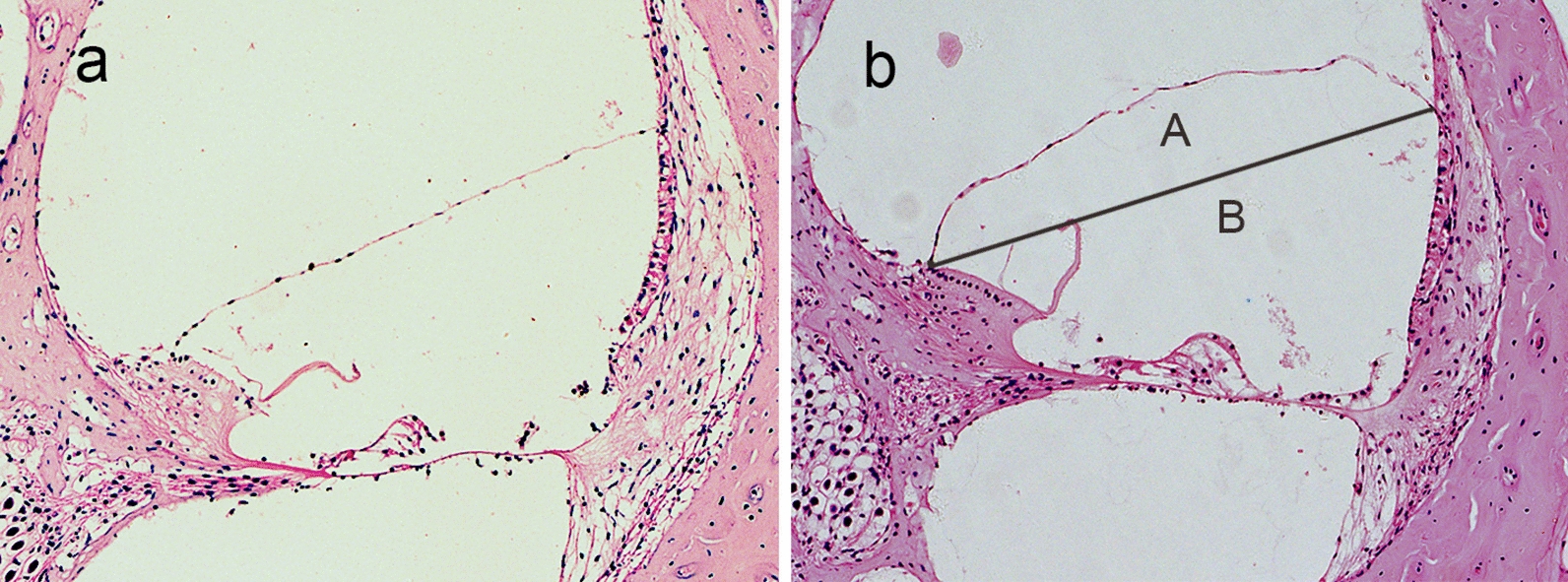
Guinea pig cochlea stained by HE. **a **The control group: normal in cochlea. **b** The treatment group: mild endolymphatic hydrops in cochlea (HE × 100). Aldosterone induced mild EH mainly in the basal turn of cochlea

Histomorphology of cochlea was detected with HE staining to assess aldosterone-induced EH. Mild EH in the basal turn was found in 9 out of 12 cochleas in the treatment group 1 month after treatment by aldosterone, and no EH was found in the control group (Fig. 1). It indicated that aldosterone can induce mild EH in a large portion of cochleas, mainly in the basal turn.

### Dot1l expression pattern in cochleas

**Fig. 2:**
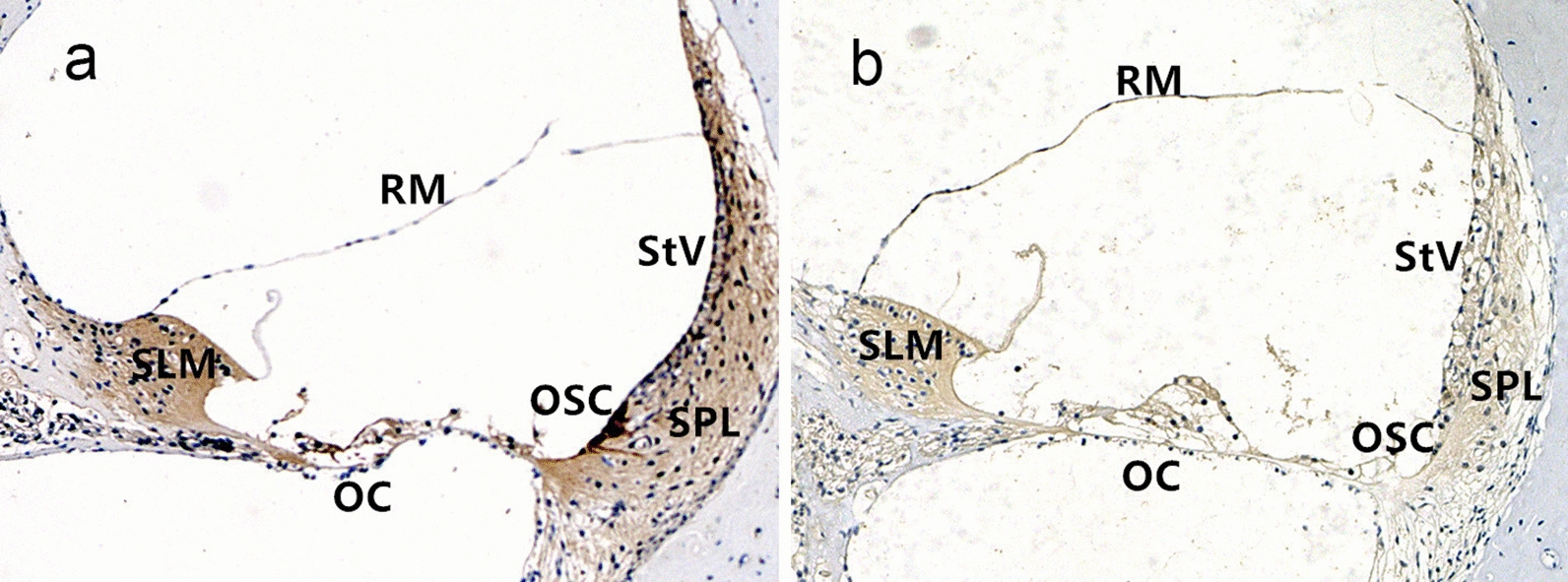
Immunohistochemical staining of Dot1l in guinea pig cochleas. **a **The control group: Dot1l expression in the stria vascularis (StV), spiral ligament (SPL), Reissner’s membrane (RM), organ of Corti (OC), spiral limbus (SLM), outer sulcus epithelial cells (OSC). **b** The treatment group: Dot1l expression in the stria vascularis, spiral limbus, spiral ligament, and organ of Corti was inhibited by aldosterone

Cellular localization of Dot1l protein in cochlea was investigated with immunohistochemistry. The results showed Dot1l expression in the stria vascularis, spiral ligament, Reissner’s membrane, organ of Corti, spiral limbus and outer sulcus epithelium cells in both groups, and Dot1l expression in the stria vascularis, spiral limbus, spiral ligament, organ of Corti was inhibited by aldosterone in the treatment group compared to that in the control group (Fig. 2).

### Total Dot1l expression in cochleas

**Fig. 3:**
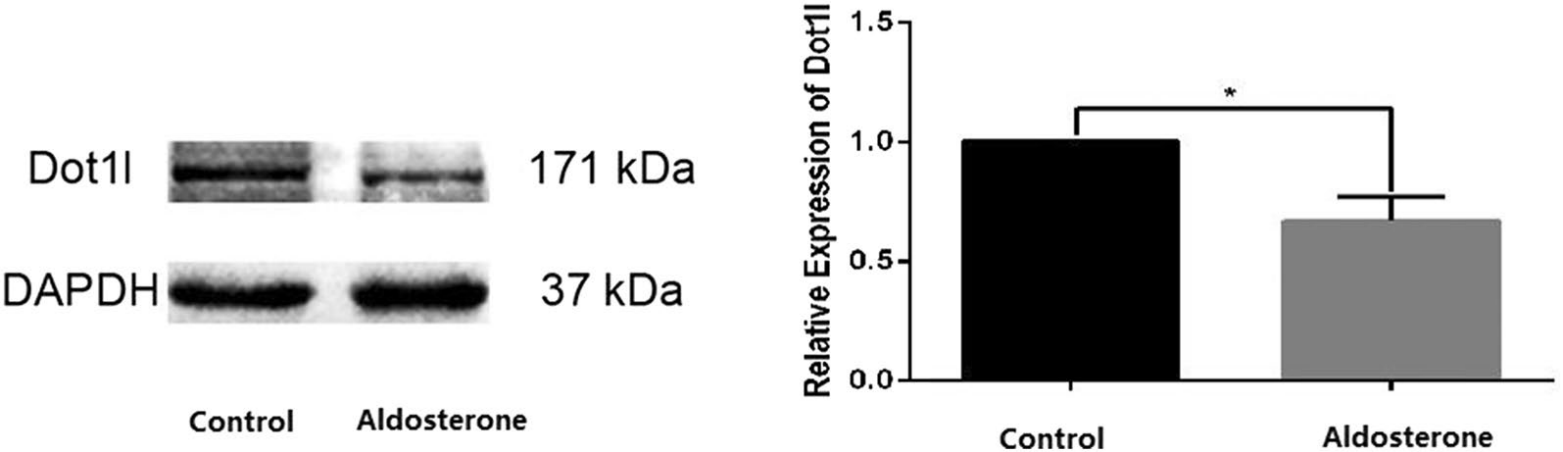
Expression levels of Dot1l in cochleas by Western blot analysis (*n* = 3 in each group). **a** Western blot bands of Dot1l protein in the cochleas of two groups. **b** The expression of Dot1l protein was lower in the treatment group than that in the control group. (*P < 0.05)

The effect of aldosterone on Dot1l expression was evaluated with Western blot. The molecular weight of Dot1l is 171 kDa, and GAPDH is 37 kDa. Dot1l expression level in the treatment group was lower than that in the control group. The expression of Dolt1l in cochleas of the treatment group normalized to GAPDH was 0.45 ± 0.04, while that of the control group was 0.30 ± 0.06 (*t* = 2.925, *P* = 0.0464) (Fig. 3), which indicated that aldosterone inhibited Dot1l expression significantly.

## Discussion

It has been well known that the pathological basis of Meniere’s disease is EH. In 1997, Dunnebier administrated aldosterone systematically following destroying the unilateral endolymphatic sac or endolymphatic duct in guinea pig. EH was induced in bilateral ears [12]. It indicates that EH may be successfully induced by intraperitoneal injection of aldosterone. In the present study, EH was found in 75% of cochleas in the treatment group, and no EH was found in the control group. Our result suggests the possible correlation between aldosterone and EH too. However, no obvious changes of hearing were found in both groups. It may be related to the possibility that ion disturbance in endolymph induced by aldosterone within 1 month is too weak to harm hair cells or spiral ganglion cells.

It has been well demonstrated that Na^+^ transporting in cochlea is mainly mediated by apically located ENaC of Reissner’s membrane and non-selective cation channels of outer sulcus cells coupled with basolaterally located Na^+^, K^+^-ATPase [13]. Na^+^, K^+^-ATPase in the basolateral membrane of epithelial cells provides the energy for Na^+^ absorption, and Na^+^ entry pathways in the apical membrane provide a gating function for Na^+^ entry from endolymph into the cytosol. Highly Na^+^-selective epithelial sodium channels (ENaCs) control sodium absorption at Reissner’s membrane, saccular extramacular epithelium, semicircular canal duct epithelium and endolymphatic sac to maintain the low concentration of Na^+^ in endolymph. When the concentration of Na^+^ in endolymph increases, the expression of ENaC is upregulated to expel the redundant Na^+^ out of endolymph. It is believed that the upregulation of ENaC is a compensation of increased Na^+^ concentration in endolymph, e.g., endolymphatic hydrops induced by aldosterone.

Aldosterone regulates ENaC expression by a classical genomic approach, i.e., binding with mineralocorticoid receptor (MR) [14]. At the early stage (within 6 h), the expression of Sgk1 is increased, and the phosphorylation of Nedd4-2 attenuates the ubiquitination of ENaC, thereby increases the expression of ENaC in cell membrane. At the late stage (1 week later), aldosterone increases protein expression through aldosterone-induced genes and proteins (AIPs). Studies on kidney have demonstrated that aldosterone inhibits the expression and interaction of Dot1a and Af9 at the late stage, weakens the hypermethylation of ENaC promoter by Dot1a–Af9 complex, and thus enhances the expression of ENaC [7]. Yao et al. have reported expression of MR in cochlea [15], and we have identified ENaC and Af9, and inhibition of Af9 by aldosterone in cochlea [2, 8]. Therefore, it is reasonable to deduce that aldosterone downregulates Af9 expression and thus upregulates ENaC expression in inner ear as it does in kidney. The present study showed that the expression of Dot1l in the cochlea was obviously reduced in cochlea after treatment by aldosterone. We therefore speculate that Dot1l as well as Af9 may be involved in sodium regulation by aldosterone in endolymph. However, it is unknown to date how Af9 and Dot1l act in regulation of ENaC by aldosterone in inner ear. Is it same as that in kidney or not? Further studies are needed to clarify the mechanism.

Moreover, the process of aldosterone-regulated ENaC expression by Dot1a at the late stage is related to Sgk1, AF17, Sirt1 and other molecules besides Af9. In kidney, aldosterone upregulates Sgk1 expression, phosphorylates Af9, and reduces the methylation of ENaC promoter by Dot1a–Af9 complex [16]. AF17 upregulates ENaC expression by competing with AF9 for binding to Dot1a and simultaneously enhancing the nucleus-to-cytoplasmic transport of Dot1a [17]. Sirt1 binds to the four subregions of the ENaC promoter which interacts with Dot1a and acts synergistically with Dot1 to downregulate ENaC transcription under basal conditions. Aldosterone inhibits Sirt1 expression and binding to ENaC promoter R0 or R1 subregion, thereby increases ENaC mRNA expression [18]. In a word, these studies have suggested that Dot1 is a crucial factor for ENaC expression in a complex regulation network. Further studies on Dot1 in inner ear are of great importance to elucidate the pathogenesis of Meniere’s disease.

The role of Dot1l in hypermethylating H3K79 has been reported not only in ENaC expression, but also in cell cycle and meiosis. Many cancers, especially mixed lineage leukemia, are related to Dot1l [19]. Phase I clinical trial of Dot1l inhibitors such as EPZ-5676, a therapeutic agent for leukemia, has been completed [20, 21]. Given the variant physiological roles Dot1l may play, the effects and adverse events of Dot1l inhibitors as therapeutic agents should be further studied.

In summary, the present paper reports the expression and regulation of Dot1l by aldosterone in cochlea for the first time. Our results indicate that Dot1l may be a crucial factor for endolymph regulation and pathogenesis of Meniere’s disease.

## Data Availability

The datasets used and/or analyzed during the current study are available from the corresponding author on reasonable request.
